# Supplementary material for: A Humanized Yeast Phenomic Model of Deoxycytidine Kinase to Predict Genetic Buffering of Nucleoside Analog Cytotoxicity
Source: Genes (Basel). 2019 Sep 30;10(10):770. doi: 10.3390/genes10100770 (PMC6826991; doi:10.3390/genes10100770)
Supplement: Supplementary file 1 [file genes-10-00770-s001.zip › Additional_Files/Additional_File7_GOTermHeatmaps.zip!/Additional_File7_GOTermHeatmaps/mitochondrial_inner_membrane.pdf]

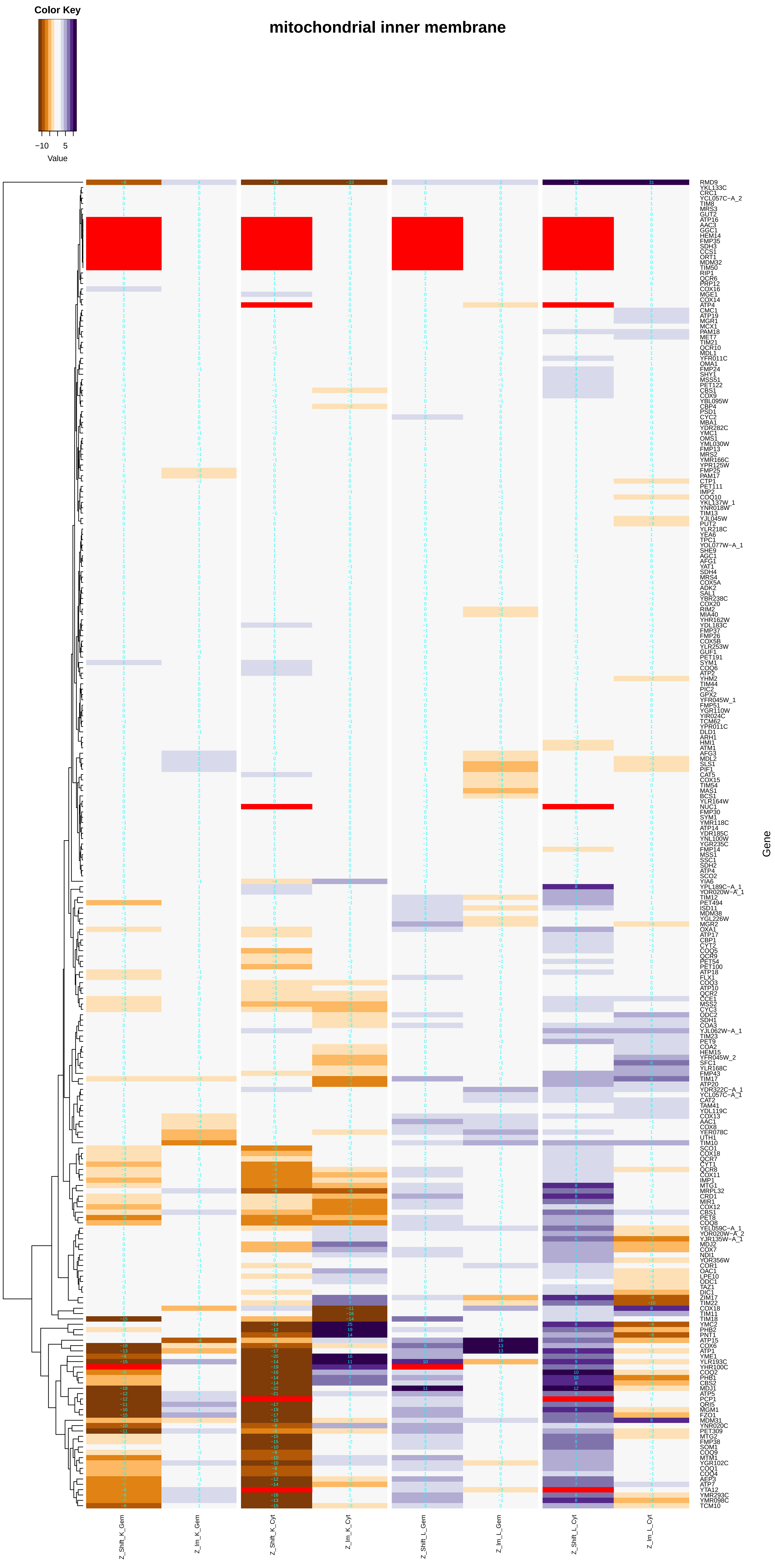
mitochondrial inner membrane
Color Key
−10
5
Value
Z_Shift_K_Gem
Z_Im_K_Gem
Z_Shift_K_Cyt
Z_Im_K_Cyt
Z_Shift_L_Gem
Z_Im_L_Gem
Z_Shift_L_Cyt
Z_Im_L_Cyt
Gene
RMD9
YKL133C
CRC1
YCL057C−A_2
TIM8
MRS3
GUT2
ATP16
AAC3
GGC1
HEM14
FMP35
SDH3
CCS1
ORT1
MDM32
TIM50
RIP1
QCR6
PRP12
COX16
MGE1
COX14
ATP4
CMC1
ATP19
MGR1
MCX1
PAM18
MET7
TIM21
QCR10
MDL1
YFR011C
OMA1
FMP24
SHY1
MSS51
PET122
CBS1
COX9
YBL095W
CBP4
PSD1
CYC2
MBA1
YDR282C
YMC1
OMS1
YML030W
FMP13
MRS2
YMR166C
YPR125W
FMP25
PAM17
CTP1
PET111
IMP2
COQ10
YKL137W_1
YNR018W
TIM13
YJL045W
PUT2
YLR218C
YEA6
TPC1
YOL077W−A_1
SHE9
AGC1
AFG1
YAT1
SDH4
MRS4
COX5A
ADK2
SAL1
YBR238C
COX20
RIM2
MIA40
YHR162W
YDL183C
FMP37
FMP26
COX5B
YLR253W
GUF1
PET191
SYM1
COQ6
ATP2
YHM2
TIM44
PIC2
GPX2
YFR045W_1
FMP51
YGR110W
YIR024C
TCM62
YPR011C
DLD1
ARH1
HMI1
ATM1
AFG3
MDL2
SLS1
PIF1
CAT5
COX15
TIM54
MAS1
BCS1
YLR164W
NUC1
FMP30
SYM1
YMR118C
ATP14
YDR185C
YNL100W
YGR235C
FMP14
MSS1
SSC1
SDH2
ATP4
SCO2
YIA6
YPL189C−A_1
YOR020W−A_1
TIM12
PET494
ISD11
MDM38
YGL226W
MGR2
OXA1
ATP17
CBP1
CYT2
COQ5
QCR9
PET54
PET100
ATP18
FLX1
COQ3
ATP10
QCR2
CCE1
MSS2
CYC3
ODC2
SDH1
COA3
YJL062W−A_1
TIM23
PET9
COA2
HEM15
YFR045W_2
SFC1
YLR168C
FMP43
TIM17
ATP20
YDR322C−A_1
YCL057C−A_1
CAT2
TAM41
YDL119C
COX13
AAC1
COX8
YER078C
UTH1
TIM10
SCO1
COX18
QCR7
CYT1
QCR8
COX11
IMP1
MTG1
MRPL32
CRD1
MIR1
COX12
CBS1
PET8
COQ8
YEL059C−A_1
YOR020W−A_2
YJR135W−A_1
MDJ2
COX7
NDI1
YOR356W
COR1
OAC1
LPE10
ODC1
TAZ1
DIC1
ZIM17
TIM22
COX18
TIM11
TIM18
YMC2
PHB2
PNT1
ATP15
COX6
ATP1
YME1
YLR193C
YHR100C
COQ2
PHB1
CBS2
MDJ1
ATP5
PCP1
ORI5
MGM1
FZO1
MDM31
YNR020C
PET309
MTG2
FMP38
SOM1
COQ9
MTM1
YGR102C
COQ1
COQ4
AEP3
ATP7
YTA12
YMR293C
YMR098C
TCM10
